# Novel TMEM173 Mutation and the Role of Disease Modifying Alleles

**DOI:** 10.3389/fimmu.2019.02770

**Published:** 2019-12-05

**Authors:** Salla Keskitalo, Emma Haapaniemi, Elisabet Einarsdottir, Kristiina Rajamäki, Hannele Heikkilä, Mette Ilander, Minna Pöyhönen, Ekaterina Morgunova, Kati Hokynar, Sonja Lagström, Sirpa Kivirikko, Satu Mustjoki, Kari Eklund, Janna Saarela, Juha Kere, Mikko R. J. Seppänen, Annamari Ranki, Katariina Hannula-Jouppi, Markku Varjosalo

**Affiliations:** ^1^Institute of Biotechnology, University of Helsinki, Helsinki, Finland; ^2^Research Programs Unit, Molecular Neurology and Biomedicum Stem Cell Centre, University of Helsinki, Helsinki, Finland; ^3^Department of Hematology and Regenerative Medicine, Karolinska Institutet, Huddinge, Sweden; ^4^Molecular Neurology Research Program, University of Helsinki and Folkhälsan Institute of Genetics, Helsinki, Finland; ^5^Department of Biosciences and Nutrition, Karolinska Institutet, Huddinge, Sweden; ^6^Faculty of Medicine, University of Helsinki, Clinicum, Helsinki, Finland; ^7^Department of Dermatology and Allergology, Skin and Allergy Hospital, Helsinki University Hospital, University of Helsinki, Helsinki, Finland; ^8^Hematology Research Unit Helsinki, Department of Clinical Chemistry and Hematology, University of Helsinki, Helsinki, Finland; ^9^Comprehensive Cancer Center, Helsinki University Hospital, Helsinki, Finland; ^10^Department of Clinical Genetics, University of Helsinki, Helsinki University Hospital, Helsinki, Finland; ^11^Department of Medical and Clinical Genetics, University of Helsinki, Helsinki University Hospital, Helsinki, Finland; ^12^Clinical Research Institute HUCH Ltd., Helsinki, Finland; ^13^Institute for Molecular Medicine Finland, University of Helsinki, Helsinki, Finland; ^14^Department of Rheumatology, Helsinki University Hospital, Helsinki, Finland; ^15^School of Basic and Medical Biosciences, King's College London, Guy's Hospital, London, United Kingdom; ^16^Rare Disease Center, Children's Hospital, University of Helsinki, Helsinki University Hospital, Helsinki, Finland; ^17^Immunodeficiency Unit, Inflammation Center, University of Helsinki, Helsinki University Hospital, Helsinki, Finland

**Keywords:** TMEM173, stimulator of interferon genes, interferon type I, IFIH1, additive effect, protein interactions

## Abstract

Upon binding to pathogen or self-derived cytosolic nucleic acids cyclic GMP-AMP synthase (cGAS) triggers the production of cGAMP that further activates transmembrane protein STING. Upon activation STING translocates from ER via Golgi to vesicles. Monogenic STING gain-of-function mutations cause early-onset type I interferonopathy, with disease presentation ranging from fatal vasculopathy to mild chilblain lupus. Molecular mechanisms underlying the variable phenotype-genotype correlation are presently unclear. Here, we report a novel gain-of-function G207E STING mutation causing a distinct phenotype with alopecia, photosensitivity, thyroid dysfunction, and features of STING-associated vasculopathy with onset in infancy (SAVI), such as livedo reticularis, skin vasculitis, nasal septum perforation, facial erythema, and bacterial infections. Polymorphism in *TMEM173* and *IFIH1* showed variable penetrance in the affected family, implying contribution to varying phenotype spectrum. The G207E mutation constitutively activates inflammation-related pathways *in vitro*, and causes aberrant interferon signature and inflammasome activation in patient PBMCs. Treatment with Janus kinase 1 and 2 (JAK1/2) inhibitor baricitinib was beneficiary for a vasculitic ulcer, induced hair regrowth and improved overall well-being in one patient. Protein-protein interactions propose impaired cellular trafficking of G207E mutant. These findings reveal the molecular landscape of STING and propose common polymorphisms in *TMEM173* and *IFIH1* as likely modifiers of the phenotype.

## Introduction

Monogenic interferonopathies are characterized by increased type I interferon (IFN) signaling leading to vasculopathy, autoinflammation, and systemic lupus erythematosus (SLE)-like disease (1). In these, increased sensing of endo- and exogenous nucleic acids leads to hyperactive Toll-like-receptors (TLRs), RNA and DNA sensors, and to an enhanced type I IFN response (2), thought to maintain the persistent self-directed immune reaction (3).

One currently known monogenic interferonopathy is STING-associated vasculopathy with onset in infancy (SAVI), caused by mutations in *TMEM173* (4, 5). *TMEM173* encodes stimulator of interferon genes (STING), a transmembrane protein residing in the endoplasmic reticulum (ER). It senses cytosolic double stranded DNA (dsDNA) and directly binds to bacterial second messengers, such as cyclic dinucleotides (CDNs) c-di-GMP, c-di-AMP, and 3′3′-cGAMP (6, 7). The recognition of nucleic acids or cyclic nucleotides initiates the production of type I IFN and other inflammatory cytokines leading to nucleic-acid driven inflammation.

STING has four amino-terminal transmembrane domains spanning the first 136 amino acids, followed by the helix α1 at residues 153-177 (8). Helix α1, or the dimerization domain, is essential for protein stability, intraprotein interactions, and ligand binding (9). The CDN binding domain (residues 153-340) is part of the cytoplasmic carboxy-terminus having multiple phosphorylation and downstream signaling interaction sites (8, 10).

The first described constitutively active *TMEM173* mutations are situated at or near the helix α1 and associated with early-onset vasculitis, autoinflammation, and interstitial lung disease defining the SAVI phenotype (5, 11, 12). A recent study identified five patients with a gain-of-function (GOF) mutation affecting the dimerization domain (13). In contrast to the severely affected infants (5) these patients presented with mild skin vasculitis and were diagnosed with familial chilblain lupus (13). Also proximal substitutions affecting the CDN binding domain were reported in single patients presenting with variable phenotypes of STING-associated autoinflammation (14, 15). Overall, all of the reported *TMEM173* mutations have been GOF, leading to elevated IFN-β production activating the JAK/STAT-pathway and generating a positive feedback loop (16). As there is poor correlation between genotype and clinical phenotype, poorly understood intrinsic or environmental factors likely modify the disease outcome.

Another important interferonopathy gene is the IFN-induced helicase C domain-containing protein 1 (*IFIH1*, also known as MDA5). IFIH1 functions as a nucleic acid sensor in the cytoplasm, inducing transcription of type I IFN and IFN-regulated genes upon activation. GOF *IFIH1* variants are seen in Aicardi-Goutières and Singleton-Merten syndromes (17–19). Also, polymorphism in *IFIH1* has been linked to SLE (rs1990760, p.Ala946Thr, A946T) (20, 21) leading to a variable phenotype spectrum (22). The A946T GOF risk variant leads to increased production of type I IFN, promoting inflammation and increasing the risk of autoimmunity. It also modifies the effects of other autoimmune risk alleles, which leads to variable disease severity (23, 24). Interestingly, a haplotype consisting of *IFIH1* T946 allele and R843 allele (rs3747517, p.His843Arg, H843R) has been reported to associate with risk of type I diabetes and psoriasis (24), but to be protective for chronic periodontitis (25).

Here we report a large family, presenting with several lupus-like features and features of SAVI (Table 1). We propose that the variable interplay of novel disease-causing G207E mutation and known polymorphism in *TMEM173* affects the disease phenotype together with *IFIH1* risk alleles. Our results broaden the spectrum of *TMEM173* mutation-associated phenotypes, provide insight into the activation of alternative NLRP3 inflammasome and reveal the STING interactome.

**Table 1:** Patient demographics and clinical features of affected family members.

**Patient**	**IV.l**	**V.2**	**III.4**	**III.9**	**III.7**	**IV.6**
**Patient demographics**						
Age of symptom onset (years)	Birth	Birth	Birth	4	8	10
Age at evaluation (years)	37	12	52	49	56	31
Gender	Male	Female	Female	Female	Female	Female
STING p.G207E	G/E	G/E	G/E	G/E	G/E	G/E
STING p.R232H rs1131769	R/H	R/H	R/H	H/H	H/H	R/H
IFIH1-risk allele p.A946T rs1990760	A/T	T/T	A/T	A/T	A/A	A/A
IFIH1-risk allele p.H843R rs3747517	R/R	R/R	R/R	R/R	H/R	H/H
**Inflammatory manifestations**						
Livedo reticularis	Widespread birth	Widespread birth	Localized birth	Widespread adulthood	Widespread 8 years	Localized early childhood
Skin vasculitis	Yes 29 years	Yes 10 years	No	No	No	No
Nasal septal perforation	Yes 29 years	Yes 6 years	No	Yes 49 years	No	No
Facial erythema	Yes	Yes	Yes	Yes	Yes	No
UV sensitivity	Burns easily	Burns easily	Burns easily	Urticaria	Burns easily	Burns easily
Alopecia at age of years	Universalis 10 years	Universalis 6 years	Areata 7 years	Totalis 4 years (grew back) and universalis 30 years	No	Universalis 10 years (scalp hair grew back) and universalis 12 years
Thyroid dysfunction	No	Hypo 12 years	Hypo 7 years	Hyper 38 years	No	Hyper 13 years
Autoimmune thyroiditis, TpoAb	No	Yes, 370 IU/mL	No, <33IU/mL	Yes, 610 IU/mL	No	Yes, 1855 IU/mL
**Infection susceptibility**						
Skin infections	Facial erysipelas 33 years, deep abscess in right thigh 27 years	Necrotizing fasciitis and abscesses in upper extremity 3 years, necrotic vasculitis and abscess in the abdomen 10 years	No	No	Abscess in left inguinum childhood	No
Periodontitis	No	No	No	No	Yes 42 years	Yes 30 years
Respiratory tract	No	Pneumonia 1.5 years	No	Recurrent sinusitis adulthood	Recurrent sinusitis adulthood	No

*Genotype phenotype associations are highlighted*.

## Materials and Methods

### Ethics, Consent, and Permissions

The study was conducted in accordance to the principles of the Helsinki Declaration and was approved by the Helsinki University Central Hospital Ethics Committee (91/13/03/00/2011). Written informed consent was obtained from the patients and healthy controls.

### Consent to Publish

Written informed consent was obtained from the patients and healthy controls (or legal guardian for child) to publish individual patient data including images.

### Patient Information

**Figure 1:**
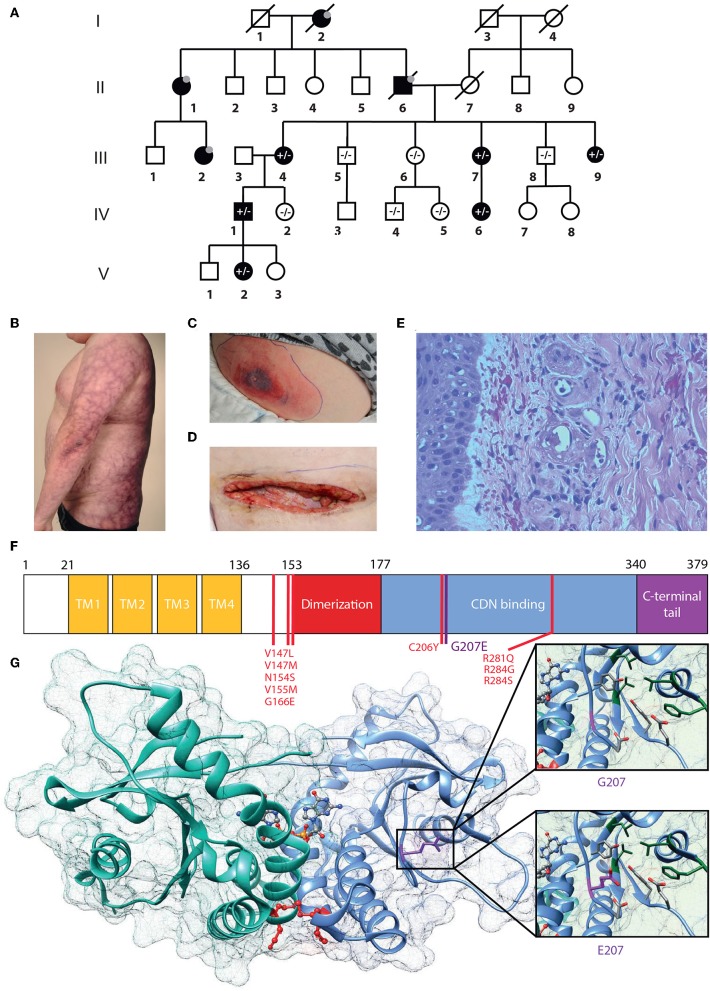
G207E STING mutation associates with SAVI and lupus-like features. **(A)** Family pedigree. Prevalence of the G207E mutant allele is shown with + and – signs, and individuals without in-depth clinical evaluation are denoted with gray dots; deceased individuals by diagonal bars. **(B)** Livedo reticularis in IV.1. **(C,D)** Necrotizing cellulitis and vasculitis in V.2 initially **(C)**, after surgical revision **(D)**. **(E)** Vasculitis in IV.1's skin with destructed vessels with neutrophils in their walls and perivascular leukocytes and erythrocytes. **(F)** STING structure showing transmembrane (TM), dimerization, and cyclic-di-nucleotide (CDN) binding domains and the carboxy-terminal tail. Previously identified mutations are denoted in red, the novel G207E mutation in purple. **(G)** Crystallographic structure of STING dimer in complex with cGAMP (PDB entry 4EMT). G207 cannot contact cGAMP or polar (red) or hydrophobic (green) residues nearby, while E207 has a polar, flexible side chain that can interact with polar amino acids and cGAMP.

From birth, the index case **IV.1** (Figure 1) has had erythema on the cheeks and severe livedo reticularis on his body and extremities, most pronounced on his sides, thighs, upper arms, hips, and buttocks. Later, remitting idiopathic thrombocytopenia and unspecified nail changes were noted at age 7 years, as well as alopecia areata. Later, alopecia universalis with sparing of genital, eyebrow and some scalp hair has developed. Repeated bone marrow aspirates and karyotype have been normal. He has nasal septal perforation and slightly dysmorphic features with hypoplastic alae nasi and deep philtrum. He is sensitive to UV radiation and burns extremely easily. He was admitted to the dermatology ward at age 29 years due to painful necrotic ulcerations resembling vasculitis on the shin and ankle that had appeared after a sunburn. Skin biopsy confirmed cutaneous vasculitis but extended laboratory tests were normal, except for an elevated Factor VIII (192–211%), considered an independent risk factor for venous thrombosis (Figure 1E). His vasculitis was first treated with prednisolone (from 0.8 mg/kg and tapered down) combined with azathioprine (150 mg, 1.5 mg/kg) or methotrexate (20 mg/week, 0.2 mg/kg), with poor response. Vasculitis then responded well to cyclosporine (maximum dose 250 mg/day, 2.5 mg/kg). A subsequent sunburn however induced re-flaring of previous ulcers. These subsided with topical corticosteroids. Additionally, he has had *Streptococcus pyogenes* periobital cellulitis and a single abscess on his inner thigh, but otherwise displays no susceptibility to infections.

A single vasculitis ulcer recurred on his lower leg at age 37 year, after the STING p.Gly207Glu mutation had been identified. In addition to a small oral prednisolone dose 0.2 mg/kg, he was started on baricitinib 4 mg/day (0.03 mg/kg) increased to 6 mg/day (0.05 mg/kg) after 2 months. The ulcer had healed after 3 months treatment. After the initiation of baricitinib his overall well-being had improved (with less fatigue and feeling ill) his hair, eyelashes, and eyebrows had started to grow and the circulation in his legs had improved. There was no visible change in the livedo, facial erythema, or laboratory parameters. He continues to receive oral baricitinib.

IV.1 has three children, of whom daughter **V.2** shares a very similar phenotype. Since birth, she has had cheek erythema and severe livedo reticularis of extremities and trunk, most severe on her thighs, upper arms, and sides. She has had UV-induced urticaria. At age 6.8 years, she developed alopecia areata, which progressed to universal alopecia with loss of all body hair except the eyebrows and eyelashes by age 7.8 years. At age 12.5 years she was diagnosed with autoimmune thyroiditis (TPOAb 370 IU/mL) and hypothyroidism. She has a nasal septal defect and similar dysmorphic features as her father. At age 3 years, she had *S. pyogenes* sepsis and necrotizing cellulitis on her upper arm and abdomen. At age 10 years, she developed an eschar on her abdomen, which developed rapidly into a large necrotic abscess with *S. pyogenes* (Figure 1C). Surgical revision of the necrotic and infected tissue produced a large wound on the abdomen (Figure 1D). Skin biopsy from the edge of the necrosis was indicative of small vessel vasculitis and underlying fasciitis. She was successfully treated with i.v. antibiotics, prednisolone (gradually tapered down from 0.5 mg/kg) and cyclosporine 100 mg/d (2.1 mg/kg). Treatment with negative pressure wound therapy successfully assisted wound closure.

From birth, the mother of IV.1 (**III.4)**, has had mild livedo reticularis on the buttocks, thighs and upper arms. At age 7 years she developed alopecia areata and was diagnosed with hypothyroidism. She burns easily in the sun and has constant facial erythema.

**III.7** developed livedo reticularis first on the buttocks at age 8 years, from where it spread to thighs and upper parts of shins, upper arms, shoulders and stomach, and most recently on her breasts. She also has facial erythema. The livedoid discoloration continuously spreads and increases. She burns easily and had UV intolerance as a child. She had early menopause at age 42 years and since then has suffered from severe periodontitis.

**IV.6** has had mild livedo reticularis form early childhood on her upper arms and knees. She burns easily in the sun. At age 10 years she developed total alopecia and, has since lacked all body hair while her scalp hair regrew within a few months. At age 12 years, total alopecia reoccurred. She has occasionally also had partial loss of eyelashes. At age 13 years, she was diagnosed with autoimmune thyroiditis and hyperthyroidism. Anti-thyroid peroxidase-antibody (TPOAb) titers reached 1,855 IU/mL at age 21 years. She was then also diagnosed with pituitary thyroid resistance and received radioiodine treatment. Thereafter, she has received thyroid hormone substitution. She has constantly elevated calcitriol and calcium levels with normal parathyroid hormone levels, not indicative of vitamin D resistance. However, after initiating calcium and vitamin D- replacement therapy, she has had minor regrowth of scalp hair. She has recurrent periodontitis and tooth enamel defects. Despite exercise-induced shortness of breath, lung function tests (spirometry and diffusion capacity) were normal.

From adulthood, **III.9** has had severe livedo reticularis, which slowly progressed on her upper arms, posterior armpits, sides, lateral back region, and buttocks extending to posterior thighs. At age 4 years, she experienced total alopecia with subsequent hair regrowth. During her childhood and adolescence, she had recurrent, intermittent spells of alopecia areata. At age 30 years, total alopecia with sparsening of eyelashes reoccurred and a vitiligo spot appeared on her cheek. At age 38 years, she was diagnosed with autoimmune thyroiditis with TPOAb levels >1,000 IU/mL. She has suffered from infertility that is unresponsive to treatment. She has a nasal septal defect, but is not sensitive to UV light. She has had recurrent sinusitis and sleep apnea, while lung function tests were normal.

### DNA Extraction

Genomic DNA was extracted from EDTA blood samples or salivary samples using Qiagen FlexiGene DNA kit (Qiagen, Valencia, CA, USA) or OraGene DNA Self-Collection Kit (OGR-250, DNA Genotek, Holliston, MA, USA).

### Genotyping and Linkage Analysis

The DNA samples of 10 family members were run on the IlluminaHumanOmniExpressExome-8v1-2 array at the Science for Life Laboratory SNP&SEQ platform in Uppsala, Sweden, according to standard protocols (Illumina, San Diego, CA, USA). Results were analyzed using Illumina GenomeStudio 2011.1 software. Two controls were run in parallel. Genotyping was based on cluster files generated from the signal intensities from more than 800 DNA samples processed in parallel to this project.

The Rutgers genetic map interpolator Rutgers Map v2 (26) was used to attain genetic map information for the markers in the linkage analysis. Merlin [v1.1.2 (27)] was used to perform parametric and non-parametric linkage analysis, using the LD-pruned genotype dataset.

### Whole Genome Sequencing

Next generation sequencing libraries were built according to established laboratory protocols at the Science for Life Laboratory Stockholm and the Institute for Molecular Medicine Finland (FIMM). The exome libraries were processed according to the Agilent SureSelect Target Enrichment System (Agilent Technologies, Santa Clara, CA, USA) for Illumina Paired-End Sequencing Libraries (Illumina, San Diego, CA, USA) using the SureSelect Human All Exon V5 capture library (Agilent Technologies, Santa Clara, CA, USA). Libraries were sequenced with 101 bp read length (HiSeq1500 sequencing platform, Illumina, San Diego, CA, USA). The read mapping, variant calling and genome annotation were performed as described previously (28). We focused the search on the haplotype that segregated with the phenotype, prioritizing rare, heterozygous coding variants that negatively affect conserved residues and shared by both whole genome sequenced individuals. Only one such variant was identified, localized within the *TMEM173* gene. The G207E variant was absent from all major public [The Exome Aggregation Consortium (ExAC), 1,000 Genomes, NHLBI Exome variant server and UK TWIN and ALSPAC study cohorts (2–4)], as well as in-house databases.

### Targeted TMEM173 PCR Amplicon Sequencing

Sample preparation was performed according to an in-house targeted PCR amplification protocol. All oligonucleotides were synthesized by Sigma-Aldrich (St. Louis, MO, USA). The protocol includes two rounds of PCR amplification. Sequencing of PCR amplicons was performed using Illumina MiSeq instrument with MiSeq Control Software v2.5 (Illumina, Inc., San Diego, CA, USA). Samples were sequenced as 251 bp paired-end reads and two 8 bp index reads. The read mapping, variant calling and genome annotation were performed as described previously (28).

### Peripheral Blood Immunophenotyping

Fresh EDTA-blood samples or peripheral blood mononuclear cells (PBMCs) were used for B and T lymphocyte immunophenotyping according to Haapaniemi et al. (28), Ilander et al. (29). Evaluation of T cell responses is also described in detail elsewhere (29).

### Site-Directed Mutagenesis Predictions

The 3D structures of STING [4LOI and 4KSY; (30, 31)] were extracted from Protein Data Bank [PDB; www.rcsb.org, (32)] and the effects on the protein stability of the individual mutants analyzed with SDM (33).

### Construct Generation

The *TMEM173* cDNA was obtained as a gateway compatible entry-clone from human Orfeome collection (Horizon Discovery, UK). All *TMEM173* variant constructs were created using Q5 Site-directed Mutagenesis Kit (New England Biolabs, Ipswich, MA, USA). The mutagenesis primers are listed in Supplementary Table IA. All the generated variant entry-constructs were confirmed with direct sequencing prior to introduction to C-MAC-Tag destination vector (34).

### Luciferase Assay

HEK293 cells were cultured in complete DMEM (Sigma-Aldrich, Espoo, Finland). 20,000 cells were seeded onto Costar 3610 white, clear bottom 96-well plates, and the following day co-transfected with 50 ng of TMEM173 constructs or empty C-MAC-tag-vector (34), 40 ng of IFN-β promoter-driven firefly luciferase reporter plasmid (IFN-β-pGL3; a kind gift from Yanick J. Crow), and 1.4 ng Renilla luciferase reporter plasmid (pRL-SV40) by using FuGENE 6 (Promega, Madison, WI, USA). Transfected cells were stimulated 24 h later with 4 μg/ml 2′3′-cyclic guanosine monophosphate (cGAMP), or 3′3′-cGAMP or c-di-GMP (all Invivogen, San Diego, CA, USA) by Lipofectamine 2000 (Thermo Fischer Scientific, Vantaa, Finland) transfection. After 24 h incubation, the cells were lysed with 1X passive lysis buffer (Promega). Luciferase assays were performed with the Dual-Glo Luciferase Assay System (Promega) according to manufacturer's instructions. For final results, counts of untransfected cells were subtracted from both firefly and Renilla luciferase, and firefly luciferase activity further normalized against Renilla luciferase activity.

### NanoString Analysis of Patient PBMCs

Blood samples were collected in Vacutainer® CPT™- tubes (BD, New Jersey, USA) and PBMCs were isolated as instructed by manufacturer. After RNA extraction, 100 ng RNA was taken for NanoString (NanoString Technologies, Seattle, USA) gene expression analysis. Our custom gene set containing in total 50 genes included 45 IFN-regulated, inflammasome-related, as well as JAK/STAT and NFκB signaling pathway genes, and five housekeeping genes. As housekeeping genes we used elongation factor 1-gamma (EEF1G), glyceraldehyde-3-phosphate dehydrogenase (GAPDH), hypoxanthine-guanine phosphoribosyltransferase (HPRT1), ornithine decarboxylase antizyme 1 (OAZ1), and tubulin beta class 1 (TUBB). For overnight hybridization at 65°C, RNAs were mixed with 5′ reporter probes (tagged with fluorescent barcodes of targeted genes) and 3′ biotinylated capture probes. Reactions were purified and immobilized on the sample cartidge surface by utilizing the Prep Station (NanoString Technologies), and the cartridge was scanned in triplicate using an nCounter Digital Analyzer (NanoString Technologies). Gene expression data was analyzed with the nSolver™ 4.0 analysis software (NanoString Technologies) performing two-step normalization using default settings as recommended by the manufacturer. The normalized values were utilized to calculate fold change for each individual gene between patient and matched control. The median fold change of all was used to calculate the interferon scores similarly to Rice et al. (35).

### Culture and Stimulation of PBMCs

PBMCs were isolated from fresh EDTA-anticoagulated blood by density gradient centrifugation in Ficoll-Paque PLUS (GE Healthcare, UK). After a minimum of 3 h rest in complete RPMI 1640 (Lonza, Basel, Switzerland) cells were stimulated with 1 μg/ml lipopolysaccharides (LPS) from *E.coli* O111:B4 (Sigma), 1 μg/ml Pam3Cys-SKKKK (Pam_3_Cys) (EMC microcollections), and 5 mM ATP (stock solution neutralized; Sigma) for the indicated times.

### Measurement of Cytokine Secretion

TNF-α and the mature, cleaved forms of IL1β and IL18 were detected from PBMC culture media supernatants using Human TNF-α DuoSet ELISA, Human IL1β/IL1F2 DuoSet ELISA, and Human Total IL18 DuoSet ELISA (all from R&D Systems).

### Quantitative Real-Time PCR

PBMC RNA was isolated using the RNeasy Plus Mini Kit (Qiagen), followed by cDNA synthesis with iScript kit (Bio-Rad). Quantitative real-time PCR was run in duplicate reactions of 10 ng cDNA using LightCycler480 SYBR Green I master (Roche Diagnostics, Espoo, Finland) and a LightCycler96 instrument (Roche). See Supplementary Table IA for primer sequences. Relative gene expression was calculated using the 2^(−ΔΔCt)^ method. The data was normalized against geometric mean of expression of two housekeeping genes (ribosomal protein lateral stalk subunit P0 and beta-2-microglobulin).

### Cell Culture

To generate stable, isogenic, inducible cell lines Flp-In™ T-REx™ HEK293 cells (Invitrogen, Life Technologies) were transfected with the generated STING-MAC-tagged constructs using FuGENE6 (Promega) as described earlier (36). Cells were cultured as instructed by manufacturer. Five 14 cm plates for each construct and biological replicate were induced with 1 μg/ml tetracycline and 50 μM biotin 24 h prior to harvesting. Harvesting, cell lysis, affinity-purification, mass spectrometry were performed as described (34, 36).

### Mass Spectrometry Filtering and Data Analysis

The mass spectrometric data was searched with Proteome Discover 1.4 (Thermo Fischer Scientific) using the SEQUEST search engine against the UniProtKB/SwissProt human proteome (http://www.uniprot.org/, version 2015-09). Search parameters were set as in Liu et al. (34). All data were filtered to high-confidence peptides according to Proteome Discoverer FDR 1%. The lists of identified proteins were conventionally filtered to remove proteins that were recognized with <2 peptides and two PSMs. The high-confidence interactors were identified using SAINT and CRAPome as in Liu et al. (34). Each sample's abundancy was normalized to its bait abundancy. These bait-normalized values were used for data comparison and visualization. The bait-normalized values from BioID data obtained from previous step were used as input for MS microscopy analysis (http://www.biocenter.helsinki.fi/bi/protein/msmic) (34). MS microscopy calculates a localization score that numerically describes the bait protein's dynamic localization in cell.

### Statistics

Statistical analysis using 2-tailed Student's *t*-test was performed using Microsoft Excel. A *p*-value less than 0.05 was considered significant.

## Results

### Clinical Presentation and Immune Phenotype

We evaluated a multigenerational Finnish family with SAVI-like features and multiple symptoms previously not associated with SAVI or chilblain lupus (Supplementary Figure 1A). Six out of ten affected family members were available for detailed clinical evaluation (Figure 1A), the main clinical findings are summarized in Table 1. Affected family members suffered from photosensitivity, alopecia ranging from areata to universalis and autoimmune thyroiditis with elevated thyroidperoxidase antibodies (TPOab) (Table 1). Prominent features included early-onset persistent livedo reticularis, skin vasculitis, recurrent, and severe infections variably reported in SAVI (Figures 1B–E). None had typical SAVI associated interstitial lung disease, violaceus facial, nasal, auricular or acral patches, ulcers or necroses, febrile attacks, failure to thrive, elevated inflammatory markers, or lupus autoantibodies (Supplementary Figure 1A; Supplementary Table IIA). The most severely affected suffered from UV induced cutaneous vasculitis, nasal septum perforation, severe infections including necrotizing cellulitis, and abscesses concomitant with SAVI (Table 1; Supplementary Figure 1A).

Immunological workup is shown in Supplementary Table II. All six evaluated patients had increased peripheral CD4+ T cell and naïve CD4+CCR7^+^CD45RA^+^ T cell count (Supplementary Table IIB). The central memory CD4+ T cells numbers remained unaffected, but pronounced decreases were observed in the effector memory subset. These observations are in accordance with findings on patients carrying activating *TMEM173* mutations (37). Among B cell populations, both naïve (CD27^−^IgD^+^) and immune-exhausted activated (CD38^low^CD21^low^) B cells were increased in 4/6 patients (Supplementary Table IIB). Acute phase reactants, blood cell counts, various autoantibodies, and complement activation were normal (Supplementary Table II).

### Identification and Structural Analysis of TMEM173 Mutation

The pedigree (Figure 1A) indicated autosomal dominant inheritance. We performed linkage analysis on 12 unaffected and 6 affected family members and whole genome sequencing on patients IV.1 and V.2. Non-parametric linkage scores of >1.7 were observed at four loci on chromosomes 3, 5, 9, and 12. We filtered the data for shared coding mutations not present in control databases (The Exome Aggregation Consortium (ExAC), 1,000 Genomes, NHLBI Exome variant server and UK TWIN and ALSPAC study cohorts (2–4), as well as in-house databases). This analysis recovered, in these four candidate loci, only one co-segregating variant which was not present in the healthy controls, was evolutionary conserved and which was predicted to damage protein structure.

This heterozygous missense mutation Chr5 (GRCh38): g.139478409C > T results in a p.Gly207Glu (G207E) substitution in STING, and was absent from major public and in-house genome databases. Using the online Combined Annotation Dependent Depletion (CADD) predictor (https://cadd.gs.washington.edu/snv) value 17 is obtained for the variant suggesting mild-to-moderate damaging effect. However, the *in-silico* predictions of the causality of mutations is extremely challenging and different prediction algorithms do rarely agree. Therefore, we set to analyze the effect of G207E more carefully using structural biology and biochemistry methods.

The protein fold of G207E mutation participates in substrate binding and STING folding. Based on protein structure prediction, G207E likely changes the electric charge of the fold, destabilizes it and potentially alters its function (Figures 1F,G, Supplementary Table IB). The G207E localizes next to a previously described mutation p.Cys206Tyr (C206Y) (14). To compare C206Y and G207E, we predicted the effects of single residue changes on protein structure and stability using Site Directed Mutator (SDM) (33). SDM predicted that both C206Y and G207E reduce the stability of the protein (pseudo ΔΔG −1.27/−1.28 and −0.72/−0.63, respectively, Supplementary Table IB) and likely lead to protein malfunction and disease. The C206Y mutation with newly acquired aromatic side chain results in more drastic structural alteration than G207E. Although the C206Y mutation received higher SDM score, the C206Y-associated phenotype is not very severe. This could be due to the limitations of the *in silico* predictions algorithms, which promotes the use of biochemical/cell biological experimentation for deciphering the causality of a mutation–and/or that addional risk alleles are involved and modulate the patient phenotypes.

### Identification of Polymorphisms in TMEM173 and IFIH1

Arginine (R) at position 232 has been identified as a major allele of human STING in the general population, albeit histidine (H) 232 is commonly listed as the wildtype allele (6). In accordance with the population data, the majority of the affected family members were heterozygotes for R232 (**Table I**). Homozygous H232 was found only in late onset livedo and recurrent sinusitis in adulthood.

A shared haplotype of two common *IFIH1* variants (rs1990760, A946T, and rs3747517 H843R) cause constitutively active IFN signaling and pre-disposition to autoimmune diseases (20, 23, 24). In the affected family four of six analyzed members (IV.1, V.2, III.4, III.9) had at least one copy of the *IFIH1* T946 risk allele, and were also homozygous for the second 843R risk variant (Table 1). In accordance to literature (25), these individuals did not have periodontitis, while the carries of A946 and H843 (III.7, IV.6) did (Table 1). Homozygosity for *IFIH1* T946 and R843 was associated with severe deep infections in V.2. Overall, based on the clinical presentations of the affected family members and their combined genotypes of the monogenic *TMEM173* mutation G207E, R232H variants, as well as the presence of the *IFIH1* risk alleles T946 and R843, we propose that *TMEM173* and *IFIH1* can jointly promote disease from 207E/H232/AA/HH toward 207E/R232/TT/RR (Table 1).

### G207E Mutation in STING Constitutively Activates IFN-β, STAT1/2, STAT3, and NFκB Transcription

**Figure 2:**
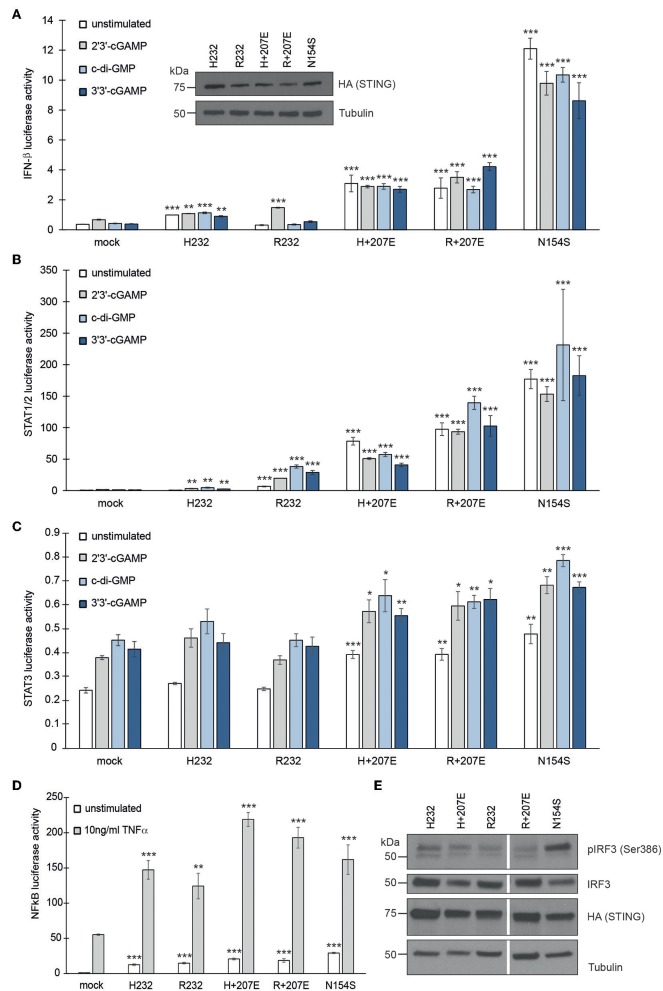
G207E constitutively activates several signal transduction pathways in HEK293 cells. Luciferase activities of **(A)** *IFN-*β, **(B)** *STAT1/2*, **(C)** *STAT3*, or **(D)** *NF*κ*B*—reporters after transient transfection with empty vector (mock), H232, R232, H + 207E, R + 207E or SAVI mutant N154S. Expression of the constructs was verified by anti-HA antibody. Through A-C, the cells were stimulated with 4 μg/ml CDNs for 24 h or left unstimulated. For *NF*κ*B* reporter assay, the cells were stimulated with 10 ng/ml TNF-α for 16 h. In all the **(A–D)** mean of minimal three replicates together with SEM is presented. Statistical significance is indicated with asterisk (*p < 0.05, **p < 0.01, ***p < 0.001; paired *t*-test). **(E)** The baseline levels of IRF3 phosphorylation remained unaltered in H232, H + 207E, R232, R + 207E. Only N154S presented elevated phosphorylation of IRF3.

To study the effect of STING mutations on activation of IFN-β, STAT1/2, STAT3, and NFκB pathways, we utilized luciferase-based reporter assays. The constitutively active STING p.Ans154Ser (N154S) SAVI mutant was used as a positive control (5). To mimic the combined genotypes of the affected family members, we combined the G207E mutation with variants R232 (genotype of patients IV.1, V.2, III.4, IV.6; herein R+207E) or H232 (genotype of patients III.9, III.7; herein H+207E). Both combinations clearly activated IFN-β, STAT1/2, and STAT3 pathways, even in unstimulated state (Figures 2A–C), thus indicating 207E as a GOF mutation.

The patients were susceptible to bacterial infections, but only a few remembered ever having viral infections. After stimulation with 2′3′-cGAMP (mimicking the mammalian response to cytosolic viral DNA) or bacterial second messengers c-di-GMP and 3′3′-cGAMP (6, 38) R+207E presented slight pathway activation in IFN-β and STAT1/2, while H+207E remained constant (IFN-β) or diminished (STAT1/2) (Figures 2A,B). These results further confirm the crucial role of R232 in cGAMP binding and IFN induction (30, 39). Both R/H+207E induced STAT3 pathway, as well as ligand-dependently hyperactivated NFκB pathway (Figures 2C,D).

We next analyzed IRF3 (Ser386) phosphorylation in stable HEK293 cells (Figure 2E). Interestingly, IRF3 phosphorylation remained at low levels in all combinations and their single counterparts, while N154S showed markedly elevated phosphorylation (Figure 2E). In concordance with our data, IRF3 independent disease development was reported in a STING N153S mouse model (40), demonstrating the involvement and activation of multiple pathways in STING-associated disease.

### Baseline Expression of Interferon Signature Genes and JAK/STAT Pathway Components Are Elevated in Patients With G207E Mutation

**Figure 3:**
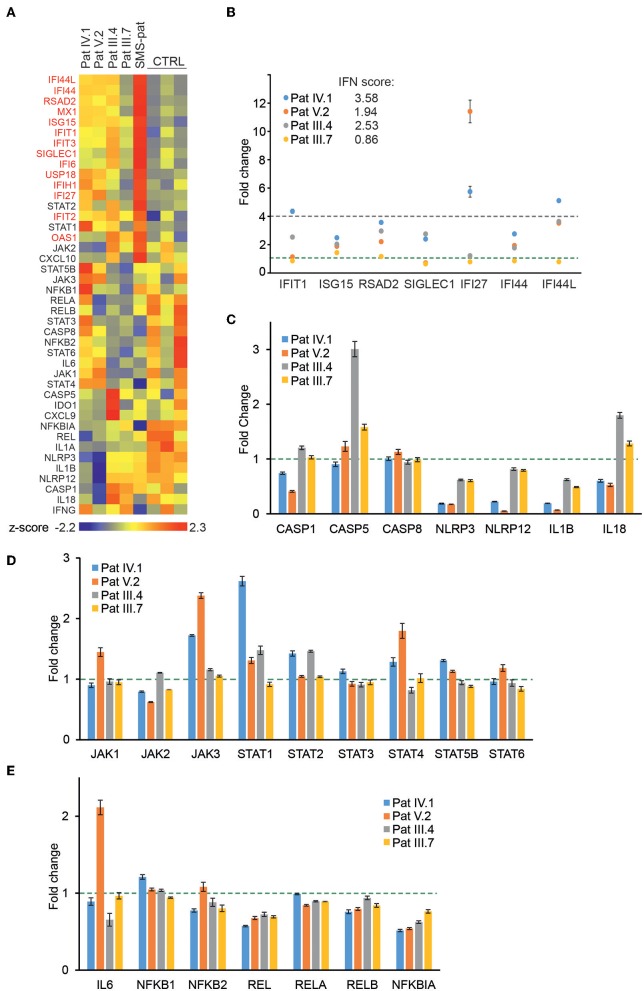
Gene expression analysis of patient PBMCs reveals alterations in IFN-regulated genes and multiple signaling pathways. **(A)** Heat map of baseline gene expression of a custom Nanostring gene set in PBMCs of four G207E patients, a Singleton-Merten syndrome patient (SMS-pat; another type I interferon-mediated disease), and three healthy controls. After background thresholding and normalization, agglomerative clustering was performed on z-scores of genes. IFN-regulated genes are marked in red. **(B)** mRNA fold changes of seven IFN-regulated genes (*IFIT1, ISG15, RSAD2, SICLEC1, IFI27, IFI44*, and *IFI44L*) and the resulting IFN score for each patient. **(C–E)** Fold changes in unstimulated patient PBMCs of inflammasome **(C)**, JAK/STAT pathway **(D)**, and NFκB pathway **(E)** related genes. Fold change 1 and 4 are indicated with green and blue dotted lines, respectively. Through **(A–E)** mean of three measurements together with SD is presented.

To directly analyze baseline gene expression in patient PBMCs, we designed a custom NanoString (41) gene set targeting multiple IFN-inducible and IFN signaling-related genes, as well as genes of the NFκB-regulated inflammasome pathway (Figure 3A). Strong upregulation of IFN-regulated genes was detected in an unrelated Singleton-Merten-patient with dominant GOF *IFIH1* mutation, validating the gene set and analysis pipeline (Figure 3A). The more severely affected G207E patients (IV.1, V.2, III.4) with both *IFIH1* risk alleles T946 and R843 displayed higher IFN-regulated mRNA levels (marked in red) than the patient (III.7) with G207E mutation and one allele of *IFIH1* R843 (Figure 3A).

For IFN signature analysis (Figure 3B) we selected seven genes (*IFIT1, ISG15, RSAD2, SIGLEC1, IFI27, IFI44*, and *IFI44L*) based on previous publications (13, 35). We noted different gene expression pattern and elevated IFN scores in patients with G207E mutation and both *IFIH1* risk alleles (Figure 3B). The patient without IFIH1 T946 and carrying one allele of R843 showed fold changes equal to healthy controls.

PBMCs displayed no clear overall tendency of baseline levels of inflammasome-related genes, whereas JAK/STAT-pathway components were modestly upregulated, and selected NFκB-related genes, including the key negative regulator NFκBIA, were downregulated (Figures 3C–E). The above speculated trend of additive effects of genetic variance was most pronounced with STING downstream targets JAK1, JAK3, STAT4, STAT6, and IL6, where patient V.2 presented the highest baseline gene expression levels (Figures 3D,E).

Baricitinib treatment clearly reduced the expression of IFN-inducible and IFN signaling-related genes in patient IV.1 (Supplementary Figure 1B). None of the other patients received immunosuppressive therapies during the time of evaluation.

### Enhanced Type I Interferon Response and Aberrant Inflammasome Activation in Patients With G207E Mutation

**Figure 4:**
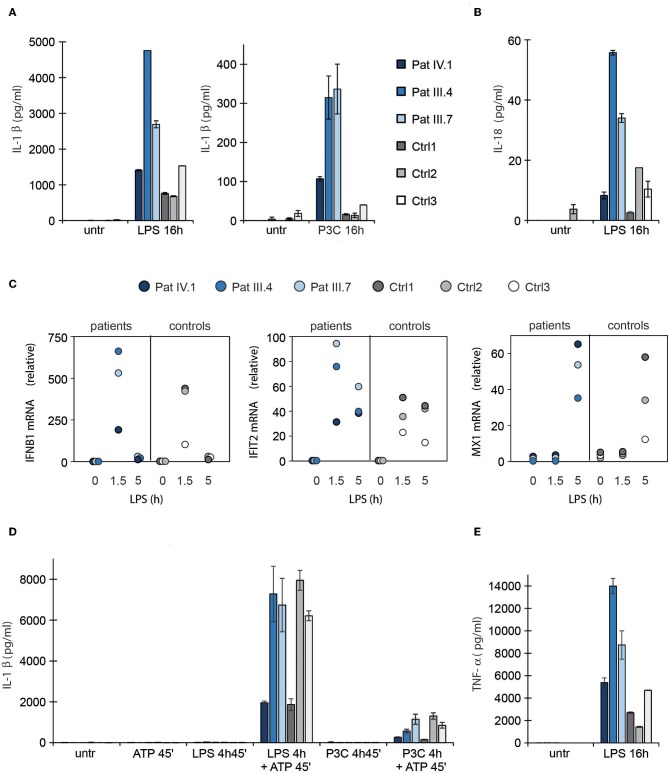
Type I interferon response and NLRP3 inflammasome activation in patient PBMCs. **(A,B)** Secretion of IL1β and IL18 in PBMC culture supernatants after activation of NLRP3 inflammasome via the alternative pathway using prolonged LPS or Pam3Cys (P3C) stimulation. **(C)** RT-PCR analysis of *IFNB1, IFIT2* and *MX1* in patient PBMCs untreated and after LPS treatment. **(D)** Secretion of IL1β in PBMC supernatant after canonical inflammasome pathway activation with LPS or Pam3Cys priming followed by ATP stimulation. **(E)** TNF-α concentration in PBMC culture supernatants after LPS stimulation. The data collection in **(A,B)** and **(D,E)** was performed with enzyme-linked immunosorbent assay (ELISA). Data are presented from three patients and three control subjects. Cytokine levels represent means and the error bars denote SD from two biological replicates per condition; for RNA isolation and qPCR, the cells from these replicates were pooled.

STING has been recently linked with activation of the NLRP3 inflammasome (42, 43), which can be activated either by the canonical, non-canonical, or alternative pathway (44). To test inflammasome hyperactivation, we stimulated patient PBMCs with LPS and a subsequent ATP pulse or with prolonged LPS alone to activate the canonical and alternative pathways, respectively. The secretion of IL1β and IL18 was used as a proxy for inflammasome activation. Pam3Cys that activates NFκB pathway via Toll-like receptor (TLR) 1/2, but does not normally trigger inflammasome signaling or type I IFN response, was used as a control (45). In the patients' PBMCs LPS induced stronger secretion of mature IL1β and IL18 via the alternative NLRP3 inflammasome pathway (Figures 4A,B) and, importantly, IL1β secretion was aberrantly triggered by Pam3Cys (Figure 4A). The NFκB-mediated induction of pro-IL1β mRNA was higher in patients' PBMCs after LPS and Pam3Cys stimulation (Supplementary Figures 2A,B), which may contribute to elevated IL1β secretion. However, only LPS but not Pam3Cys triggers signaling via TRIF adapter molecule that is required for activation of alternative NLRP3 inflammasome pathway and subsequent proteolytic maturation of IL1β/IL18 (45). This suggested that the enhanced cytokine secretion in patients' PBMCs was primarily driven by altered signaling rather than elevated mRNA levels. As the patients' PBMCs showed normal IL1β/IL18 responses to ATP stimulation after short priming (**Figure 4D**; Supplementary Figure 2C), the mutant STING mainly targeted alternative NLRP3 inflammasome signaling.

LPS also induced a stronger type I IFN response in the patients' PBMCs (Figure 4C). In line with this, alternative NLRP3 inflammasome pathway and type I IFN response both utilize a TRIF-dependent signaling pathway downstream LPS (45). Conceivably, constitutive type I IFN activity driven by mutant STING could involve activation of a signaling molecule shared with the alternative NLRP3 inflammasome pathway, enabling also Pam3Cys-triggered IL1β secretion. The patients also showed elevated TNF-α response to LPS, confirming a wide dysregulation of TLR responses, including NFκB (Figure 4E; Supplementary Figure 2B). However, we did not see a general correlation between clinical disease severity and cytokine secretion (Figures 4A,B,E).

### Molecular Context of Mutant STING

**Figure 5:**
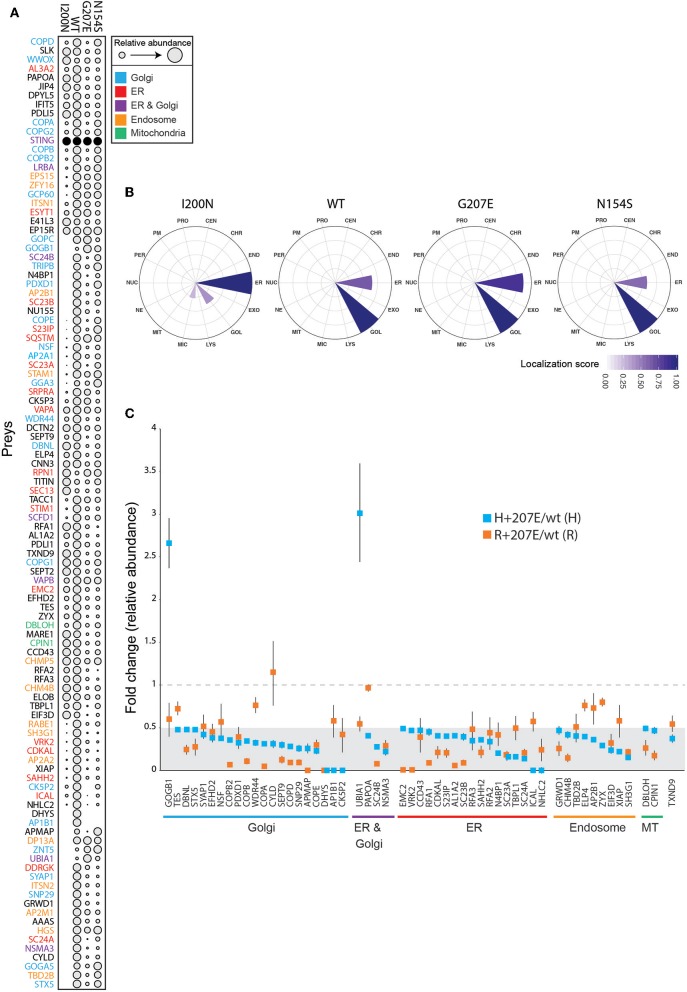
Functional data of STING wt and mutant cell lines shows differences in genotypes. **(A)** Bait expression normalized relative amount of identified interacting proteins (preys) in stable I200N (null mutant), H232 (wt), G207E, and N154S 293 cells. Dot size indicates relative prey abundance. **(B)** Mass spectrometry microscopy assigned localizations at steady state. Localization abbreviations denote the following compartments: peroxisome (PER), microtubule (MIC), endosome (ED), proteasome (PRO), nuclear envelope (NE), Golgi (GOL), lysosome (LYS), nucleolus (NUC), plasma membrane (PM), endoplasmic reticulum (EM), mitochondria (MIT), centrosome (CEN), chromatin (CHR), and exosome (EXO). Color gradient indicates localization score. **(C)** Identified proteins and their fold changes in H + 207E and R + 207E compared to their wild-type counterparts. The light gray area indicate fold change 0–0.5, and dotted line fold change equal to 1.

To characterize the molecular context of STING at near physiological levels (46), we analyzed the protein-protein interactions of H232 (wild-type; wt), I200N [null mutant (47, 48)], N154S (5), and H+207E in inducible HEK293 cells using BioID proximity labeling (49). The BioID-method tags the protein of interest (bait) with a modified biotin ligase, which adds biotin to the closely interacting proteins (preys) (49). The biotinylated proteins are then affinity-purified and analyzed with quantitative mass spectrometry. In line with previous data (39), the wt STING preferentially interacted with Golgi, ER, endosomal and mitochondrial proteins (Figure 5A). I200N, G207E, and N154S lost multiple interactions compared to the wt. Hierarchical clustering of the interactions of the mutants form a continuation from the null allele (I200N) to GOF SAVI mutant (N154S), showing G207E between the wt and the N154S (Figure 5A). This result is consistent with the reporter assay data and milder clinical phenotype in G207E carriers (Figure 2).

We utilized mass spectrometry microscopy (MS microscopy) (34) on our steady state BioID data. The resolution of MS microscopy outperforms fluorescent microscopy in refining the subcellular location (34), and showed I200N localization mainly to ER, while G207E, N154S, and wt were predominantly localized to Golgi (Figure 5B). Fascinatingly, the G207E localized more to the ER than wt or N154S (localization scores 0.86, 0.66, and 0.58, respectively; Figure 5B). It could be speculated that G207E mutant protein induces ER stress and is more rapidly degraded than WT or N154S STING –however, this is not visible in the WB- or MS analyses of the patients' PBMCs or transgenic HEK293 cell lines expressing the different STING mutants, respectively (Supplementary Figures 3A,B).

To gain in-depth information of G207E, we analyzed its interactome in both allelic backgrounds (H/R + 207E). Generally, the G207E mutation led to a reduction of protein-protein interactions with the proteins residing in Golgi, ER, endosome and mitochondria, compared to the corresponding wt (Figure 5C). Notably, the G207E mutant had weak interactions with all components of the coatomer complex I (COPI)-machinery mediating retrograde transport from Golgi to the ER (50, 51) (Figures 5A,C). Also, multiple components of the coatomer complex II responsible for ER to Golgi transport [syntaxin 5 (STX5)], protein transport protein Secs (Sec23A, Sec23B, Sec24A, Sec24B) were downregulated (Figures 5A,C). The interactions between GOGB1 and UBIA1 were stronger in the H+207E mutant (>two-fold). UBIA1 localizes to ER-Golgi compartment, while GOGB1 mainly resides at the cis-Golgi participating in COPI-mediated transport (52–54). Comparison of interactions between R+207E and H+207E showed clear differences in five genotype-specific interactions (Supplementary Figure 3C). R+207E lost association with APMAP (adipocyte plasma membrane-associated protein with uncharacterized function), but interacted more with PDXD1 (pyridoxal-dependent decarboxylase domain-containing protein 1) and RFA1 (Replication protein A 70 kDa DNA/binding subunit) compared to H + 207E.

These data suggest that in addition to amplifying downstream signaling, the STING GOF mutations cause broad alterations in subcellular localization and plausibly defective vesicular trafficking. The features overlap with other known monogenic autoimmune diseases that are caused by defects in the vesicular trafficking (55, 56). The alterations likely contribute to the autoimmune and infectious manifestations seen in the affected patients.

## Discussion

We report a large family with a distinct somewhat lupus-like and SAVI-like phenotype carrying a novel G207E mutation in the substrate binding domain of STING. We propose that the variance in disease severity in this family results from a combinatory additive effect of coding polymorphisms in *TMEM173* and *IFIH1* genes. The disease retained some typical characteristics of SAVI e.g., cutaneous vasculitis, severe infections including necrotizing cellulitis, exacerbation of skin symptoms by cold and nasal septum defects (5, 11, 14), but was considerably milder and lacked early onset lung manifestations, violaceus, and ulcerative skin lesions, febrile attacks and elevated inflammatory and autoinflammatory markers (Supplementary Figure 1A). Instead, our patients presented with several previously unreported STING-associated lupus-like features including alopecia, photosensitivity, and thyroid dysfunction. Most of them had marked and widespread livedo reticularis from birth. Furthermore, their IFN score was less severely elevated.

Consistent with SAVI patients (5, 37), all our patients had expanded population of naïve CD4+ and CD8+ T-cells but low numbers of mature B and T cells. STING has also been proposed to act as an inhibitor of immune cell proliferation (37), an effect linked to NFκB activation and spatially limited to Golgi (37). This finding is supported by our data, which shows hyperactivation of NFκB in the patient cells, and also an altered localization of the G207E mutant within the Golgi/ER compartment.

As there is considerable variety in disease severity among the *TMEM173* mutant carriers (5, 11, 13–15), we further analyzed the effect of common polymorphisms in *TMEM173* (p.R232H) and *IFIH1* (p.A946T and p.H843R) on disease phenotype. As note, all genotyped patients were negative for the HAQ allele (R71H-G230A-R293Q). Patients with the R232 variant had more severe symptoms. *In vitro*, this polymorphism strengthened the enhanced activation of the mutant STING, leading to the overexpression of STING downstream targets IFN, IL1β, and IL18. The observation is consistent with previously reported effect of R232 on amplifying IFN-β pathway activation *in vitro* (6). We also noted a trend of additive clinical effect for the G207E mutant and the three polymorphisms, with the combination R232+G207E+IFIH1 T/T+R/R leading to a severe early-onset phenotype that resembles the SAVI-features (Pat V.2). These *IFIH1* risk alleles have been shown to promote disease through a combinatorial effect (24). Although supported by experimental data, the effects need validation in larger patient sets.

Understanding of the crosstalk between STING-pathway and the NLRP3 inflammasome controlling IL1β and IL18 secretion is expanding, and recently STING was shown to have a role in inflammasome activation (42, 43). We observed aberrant alternative NLRP3 inflammasome activation in response to bacterial TLR stimuli in the patient PBMCs. The constitutively active STING-signaling in our patients' PBMCs could explain the elevated LPS-induced IL1β secretion and drive the observed aberrant inflammasome activation utilizing an adaptor protein TRIF (45, 57). Indeed, LPS was reported to aberrantly induce IL1β secretion in STING V155M expressing *TRIF*^−/−^ monocytic cells (42). Activation of NLRP3 inflammasome has a key role in the pathogenesis of other autoinflammatory diseases, such as cryopyrinopathies (58). Our data suggests that dysregulation of both interferons and IL1β may contribute to the disease phenotypes of interferonopathies.

To our knowledge, our study is the first to define the molecular interactions of STING. The majority of STING partners were Golgi, ER or endosomal proteins, consistent with reports of subcellular localization of STING (8, 39). The monogenic GOF mutants N154S and G207E led to reduction of interactions. Striking difference between N154S and G207E are the weakened or lost interactions of G207E with COPI and COPII complexes, essential for protein transport between ER and Golgi (59). Loss of COPI also causes monogenic autoimmune disease (56). The increased G207E interactions included Sequestosome-1 (SQSTM) an autophagy receptor involved in endosome organization (60). The combined loss of COPI and gained SQSTM interactions together with aberrant subcellular localization suggest impairment in mutant STING trafficking. Since STING has been shown to predominantly signal from endosomes, the impaired trafficking likely leads to prolonged signaling and activation of downstream pathways (59, 61). Interaction with AP-1 complex subunit beta-1 (AP1B1), a protein involved in cargo sorting at trans-Golgi network, was lost or diminished in both H + 207E and R + 207E, further supporting the model of defective transport.

The interactomes of G207E in polymorphic backgrounds (H232 and R232) were highly similar. The R + G207E variant, which is associated with severe clinical disease, further diminished the G207E interactions with the COPI proteins—likely exaggerating the trafficking defect, and further prolonging STING signaling from the endosomes. R + 207E variant also associated more with ubiquitin carboxyl-terminal hydrolase CYLD, which regulates inflammation and immune responses via NFκB-inducing pathways (62–64).

To conclude, we describe in a multigenerational family a novel STING mutation G207E as a cause for a combination of SAVI-associated vasculopathy and selected lupus-like features. We suggest that *TMEM173* polymorphism p.R232H as well as *IFIH1* autoimmune risk variants p.A946T and p.H843R might act as cumulative disease modifiers contributing to the disease severity. However, more patients are needed to further investigate this phenomenon. Treatment with JAK1/2 inhibitor baricitinib was beneficiary in one patient. We also describe the interactomes of wt and mutant STING, demonstrating a localization defect in the latter, and report elevated NRLP3 inflammasome activation and IL1β secretion in patient PBMCs, potentially contributing to the disease phenotype. The results broaden the clinical spectrum of STING mutation phenotypes, and increase understanding of the molecular mechanisms of nucleic acid-driven inflammatory diseases.

## Data Availability Statement

The raw data supporting the conclusions of this manuscript will be made available by the authors, without undue reservation, to any qualified researcher.

## Ethics Statement

The studies involving human participants were reviewed and approved by Helsinki University Central Hospital Ethics Committee (91/13/03/00/2011). Written informed consent to participate in this study was provided by the participants' legal guardian/next of kin. Written informed consent was obtained from the individual(s), and minor(s)' legal guardian/next of kin, for the publication of any potentially identifiable images or data included in this article.

## Author Contributions

SKe, EH, EE, and KR designed and performed experiments, analyzed data, wrote the manuscript, and prepared the figures. HH, MI, MP, EM, KH, and SL performed experiments. SKi, SM, KE, JS, and JK supervised experiments and data analysis. MS, AR, KH-J, and MV designed experiments and wrote the manuscript.

### Conflict of Interest

KH was employed by the Clinical Research Institute HUCH Ltd. of The University of Helsinki Hospital, Helsinki, Finland. MS has received honoraria from CSL Behring. SM has received honoraria from BMS and Novartis. JS has received honoraria from Roche. KH-J has received honoraria from Octapharma and Abbvie. AR is an Advisory Board Member of ImmunoQure Gmbh. The remaining authors declare that the research was conducted in the absence of any commercial or financial relationships that could be construed as a potential conflict of interest.

## Abbreviations

2′3′-cGAMP, cyclic GMP-AMP: mammalian cGAMP (tlrl-nacga23)
3′3′-cGAMP, cyclic GMP-AMP: bacterial cGAMP (tlrl-nacga)
cGAS: cyclic GMP-AMP synthase
c-di-GMP: cyclic di-guanylate monophosphate (tlrl-nacdg)
CDN: cyclic-di-nucleotide
FCL: familial chilblain lupus
GOF: gain-of-function
IFIH1: interferon induced with helicase C domain 1
SAVI: STING-associated vasculopathy with onset in infancy
SLE: systemic lupus erythematosus
SMS: Singleton-Merten syndrome
STING: Stimulator of interferon genes protein
TMEM173: transmembrane protein 173.
