# Supplementary material for: Novel TMEM173 Mutation and the Role of Disease Modifying Alleles
Source: Front Immunol. 2019 Dec 5;10:2770. doi: 10.3389/fimmu.2019.02770 (PMC6907089; doi:10.3389/fimmu.2019.02770)
Supplement: Supplementary file 1 [file Table_1.pdf]

SUPPLEMENTARY TABLE I

A

**Mutagenesis primers** (F, forward primer; R, reverse primer)	
**Primer**	**Sequence 5' -> 3'**
TMEM173_N154S_F	GGAATTTCAGCGTGGCCCATGGGCTGGCAG
TMEM173_N154S_R	ATGGGCCACGCTGAAATTCCCTTTTTCACACACTGCAG
TMEM173_I200N_F	CGGCTGTATAATCTCCTCCCATTGGACTGTG
TMEM173_I200N_R	TGGGAGGAGATTATACAGCCGCTGGCTCACT
TMEM173_G207E_F	TTGGACTGTGAGGTGCCTGATAACCTGAGTATGGC
TMEM173_G207E_R	ATCAGGCACCTCACAGTCCAATGGGAGGAGTTATATG
TMEM173_H232R-F	CAGCAGACCGGTGACCGTGCTGGCATCAAGGATCGGG
TMEM173_H232R-R	GATGCCAGCACGGTCACCGGTCTGCTGGGGCAGTTT

**Quantitative real-time PCR primers** (F, forward primer ; R, reverse primer)		
**Primer**	**Sequence 5' -> 3'**	**Gene**
ASC_F	TTGGACCTCACCGACAAGC	apoptosis-associated speck like protein containing a CARD domain
ASC_R	ATGTCGCGCAGCACGTTA	
B2M_F	GAGTATGCCTGCCGTGTGAA	beta-2 microglobulin (housekeeping)
B2M_R	TGCGGCATCTTCAAACCTCC	
CASP1_F	ATCCCACAATGGGCTCTGTTT	caspase-1
CASP1_R	CTCTTTCAGTGGTGGGCATCT	
IFIT2_F	GCCGAACAGCTGAGAATTGC	interferon induced protein with tetratricopeptide repeats 2
IFIT2_R	AGGCCAGTAGGTTGCACATTG	
IFNB1_F	GAGCTACAACTTGCTTGGATTCC	interferon beta 1
IFNB1_R	CAAGCCTCCCATTCAATTGC	
IL1B_F	TGGCAATGAGGATGACTTGT	interleukin-1 beta
IL1B_R	GGAAAGAAGGTGCTCAGGTC	
IL18_F	TCAACTCTCTCCTGTGAGAACAAA	interleukin-18
IL18_R	GTCCTGGGACACTTCTCTGAAA	
MX1_F	CGAGATGTCCCGGATCTGACT	MX dynamin like GTPase 1
MX1_R	CACCACCAGGCTGATTGTCT	
NLRP3_F	CAACTGCAACCTCACGTCAC	NLR family pyrin domain containing 3
NLRP3_R	ACGGTCAGCTCAGGCTTTTC	
RPLP0_F	GAAATCCTGAGTGATGTGCAGC	ribosomal protein lateral stalk subunit P0 (housekeeping)
RPLP0_R	TCGAACACCTGCTGGATGAC	
TNF_F	TGCTGCACTTTGGAGTGATCG	tumor necrosis factor alpha
TNF_R	ATCTCTCAGCTCCACGCCATT	

B

**SDM predictions on STING protein stability with C206Y and G207E patient mutations**										
**Mutation**	**PDB File**	**Residue 232**	**WT-RSA (%)**	**WT-DEPTH (Å)**	**WT-OSP**	**MUT-RSA (%)**	**MUT-DEPTH (Å)**	**MUT-OSP**	**Predicted pseudo ΔΔG**	**Outcome**
**C206Y**	4LOI	H	0.0	7.4	0.56	0.0	8.0	0.7	-1,27	Reduced stability
**G207E**	4LOI	H	57.1	4.4	0.35	49.8	3.5	0.27	-0,72	Reduced stability
**C206Y**	4KSY	R	0.0	6.7	0.53	0.1	7.0	0.64	-1,28	Reduced stability
**G207E**	4KSY	R	82.5	3.9	0.32	73.6	3.3	0.23	-0,63	Reduced stability

Percentage relative sidechain solvent accessibility (RSA) for wildtype (WT) and both mutant (MUT) residues at 206 and 207. Residue depth is presented for WT and MUT residues in angstrom units (Å). Residue occluded surface packing (OSP) is likewise presented for WT and MUT. Predicted pseudo ΔΔG and its effect on protein stability (outcome) are presented.
