# Supplementary material for: Novel TMEM173 Mutation and the Role of Disease Modifying Alleles
Source: Front Immunol. 2019 Dec 5;10:2770. doi: 10.3389/fimmu.2019.02770 (PMC6907089; doi:10.3389/fimmu.2019.02770)
Supplement: Supplementary file 2 [file Table_2.pdf]

SUPPLEMENTARY TABLE II

A Blood counts, autoantibodies and functional assay findings in STING G207E carriers.

Patient	Control Range	IV.1	V.2	III.4	III.7	III.9	IV.6
**Leukocytes** (10^6/l)	3400-8200	4400	5100	4500	5900	5600	4200
**Lymphocytes** (10^6/l)	1300-3600	2240	1880	1600	2180	1860	1270
**Monocytes** (10^6/l)	200-800	190	110	540	500	220	100
**Neutrophils** (10^6/l)	1500-6700	1820	2840	2190	3040	3200	2580
**Basophils** (10^6/l)	0-100	20	10	10	10	10	0
**Eosinophils** (10^6/l)	30-440	120	270	180	120	300	100
**B cells (CD19+)** (10^6/l)	100-500	70	340	180	180	290	50
**CD3+CD4+** (10^6/l)	300-1400	802	929	869	1065	1030	712
**CD3+CD8+** (10^6/l)	200-1200	290	310	250	600	430	210
**NK cells (CD3$^-$CD16$^+$56$^+$)** (10^6/l)	90-600	50	180	180	280	120	200
**Platelets** (10^6/l)	150 000-360 000	107 000	254 000	229 000	279 000	274 000	173 000
**ANA-ab** (titer)	<320	<80	<80	<80	<80	<80	<80
**ENA-ab**	Negat	Negat	Negat	Negat	Negat	Negat	Negat
**DNA-ab** (IU/ml)	<10	<10	<10	NA	<10	<10	<10
**ANCA**	Negat	Negat	Negat	Negat	NA	Negat	NA
**LupusAK**	Negat	Negat	Negat	NA	Negat	Negat	NA
**PLAb**	Negat	Negat	NA	NA	Negat	NA	NA
**KardAbG** (U/ml)	<15	<4	<4	NA	<4	4	NA
**IgE** (kU/l)	0-110	290	4418	738	889	46	889
**Complement**	Classical, alternative, mannan-binding lectin pathway hemolytic activities	Normal	Normal	Normal	Normal	Normal	Normal
**Lymphocyte proliferative responses to mitogens**	Phytohemagglutinin, concanavalin A, pokeweed mitogen	Normal	Normal	NA	NA	Normal	NA

B Hematologic parameters of T- and B-cells of affected family members.

Patient		Control Range (%)	IV.1	V.2	III.4	III.7	III.9	IV.6
	CD4-CD8-TCRab+	0,3-3,3	0.6	3.1	0.2	0.7	0.1	0.3
	TCRgd+	1,9-11,7	0.4	2.8	2.5	3.0	1.1	2.0
**CD3+CD4+ T cells**		35.6–56.0	73.6	72.6	77.6	63	73.9	74.9
**Naïve**	CCR7$^+$CD45RA$^+$	20.5-54.8	62.8	69.3	71.3	59.9	61.3	76.1
**TCM**	CCR7$^+$CD45RA$^-$	8.4-32.8	30.2	19.6	20.9	27.8	24.8	14.6
**TEM**	CCR7$^-$CD45RA$^-$	19.9-52.4	6.3	7.9	6.7	11.3	11.3	8.5
**TEMRA**	CCR7$^-$CD45RA$^+$	1.4-17.0	0.7	1.7	1.0	1.0	2.6	0.8
**Activated**	HLADR$^+$CD38$^-$	2.4-9.6	4.5	4.3	8.4	7.4	7.0	3.6
	HLADR$^-$CD38$^+$	40.4-72.9	62.8	74.7	70.3	50.7	53.5	77.0
	HLADR$^+$CD38$^+$	0.9-4.6	3.3	3.9	5.0	3.9	2.0	1.9
**RTE**	CD45RA$^+$CD62L$^+$CD31$^+$	14.4-38.3	28.8	43.2	28.6	16.7	19.0	24.5
**Treg**	CD25^{hi}CD127^{lo}	NA	9.8	8.6	6.6	7.3	5.8	6.5
**CD3+CD8+ T-cells**		13.1–34.5	26.6	24.2	22.3	35.7	30.9	22.1
**Naive**	CCR7$^+$CD45RA$^+$	18.8-71.0	45.3	68.9	32.6	23.0	27.8	53.6
**TCM**	CCR7$^+$CD45RA$^-$	1.2-7.3	22.8	4.9	7.1	14.7	15.1	6.6
**TEM**	CCR7$^-$CD45RA$^-$	14.6-63	7.8	5.9	12.7	16.8	10.1	16.2
**Temra**	CCR7$^-$CD45RA$^+$	4.5-33.7	24.1	20.3	47.5	45.5	47.0	23.6
**Activated**	HLADR$^+$CD38$^-$	3.8-32.4	13.7	14.9	33.2	23.8	22.4	5.0
	HLADR$^-$CD38$^+$	30.3-78.5	19.3	50.1	15.1	15.1	11.6	39.1
	HLADR$^+$CD38$^+$	1.4-21.4	12.0	12.8	24.5	14.0	13.5	6.3
		0-110 kU/l	290	4418	738	889	46	889
**CD19+ B- cells**		5-22	3	15	9	9	15	3
**Naïve**	CD27$^-$IgD$^+$	43,2-82,4	31.6	89.9	86	91.6	86.2	60.6
**Activated**	CD38^{low}CD21^{low}	0.6-3.5	12.4	6	7.5	2.8	1.5	9.1

Negat= negative, NA = not available. Values higher than control range are highlighted with red, whereas lower values are indicated with blue background.
