# Supplementary material for: Novel TMEM173 Mutation and the Role of Disease Modifying Alleles
Source: Front Immunol. 2019 Dec 5;10:2770. doi: 10.3389/fimmu.2019.02770 (PMC6907089; doi:10.3389/fimmu.2019.02770)
Supplement: Supplementary file 3 [file Data_Sheet_1.docx]

**Supplementary Material to:**

**Novel TMEM173 mutation and the role of disease modifying alleles**

Salla Keskitalo PhD^1^, Emma Haapaniemi MD, PhD ^2, 3X^, Elisabet Einarsdottir PhD^4, 5,X^, Kristiina Rajamäki PhD^6^, Hannele Heikkilä MD, PhD^7^, Mette Ilander PhD^8^, Minna Pöyhönen MD, PhD^9^, Ekaterina Morgunova PhD^3^, Kati Hokynar PhD^10^, Sonja Lagström MSc^11^, Sirpa Kivirikko MD, PhD^9^, Satu Mustjoki MD, PhD^8^, Kari Eklund MD, PhD^6,12^, Janna Saarela MD, PhD^11^, Juha Kere MD, PhD^4,5,13^, Mikko Seppänen MD, PhD^14,15*,^ Annamari Ranki MD, PhD^7^, Katariina Hannula-Jouppi MD, PhD, MBA^4,7*^, and Markku Varjosalo PhD^1*^

**Table of Contents**

**Table SI**: A) Mutagenesis and quantitative real-time PCR primers. B) Predictions on STING stability with C206Y and G207E site mutagenesis.

**Table SII**: A) Blood counts, autoantibodies and functional assay findings in STING G207E carriers. B) Hematologic parameters of T- and B-cells of affected family members

**Figure S1**: A) Comparison of clinical phenotypes in G207E mutation carriers and in previously reported SAVI and chilblain lupus patients. B) NanoString gene expression analysis after baricitinib treatment

**Figure S2**: Inflammasome-related gene expression and canonical NLRP3 pathway activation

**Figure S3:** Effect of 207E on protein stability and functions

**Supplementary figure legends**

**Supplementary Figure 1:** A) Comparison of clinical phenotypes in G207E mutation carriers (n=6) and in previously reported SAVI (n=21) ^1-9^ and chilblain lupus patients (n=5) ^10^. * Pneumonia, cellulitis, necrotizing fasciitis, septicemia; ** Antinuclear 54%, antiphospholipid 19%, ANCA 15%, cardiolipin 4%. B) NanoString gene expression analysis after baricitinib treatment. Gene expression analysis of patient PBMCs reveals normalization of IFN-regulated genes and JAK/STAT signaling pathway upon baricitinib treatment. Patient IV.1 received oral baricitinib for 1 month prior to sampling. Whereas the SMS-patient was treated for 3 months.

**Supplementary Figure 2:** Inflammasome-related gene expression and canonical NLRP3 pathway activation. A-B**)** Inflammasome-related mRNA expression levels, and C) amount of mature IL18 in PBMC culture supernatant after activation of canonical NLRP3 pathway by short TLR priming followed by 45 min ATP stimulation.

**Supplementary Figure 3:** Effect of 207E on protein stability and functions

A) STING expression levels from patients´ PBMC cells visualized by WB using TMEM173 antibody (ab92605). B) Effect of 232 R or H allele and 207 G or E allele on the STING stability and abundance, measured by corresponding H and R allele specific tryptic peptide from transgenic HEK293 cell lines with the use of mass spectrometry. C) Identified proteins and their fold changes of R+207E to H+207E reveal genotype specific protein-protein interactions. The light grey areas indicate fold change 0-0.5, and dotted line fold change equal to 1.

**REFERENCES**

1. Jeremiah N, Neven B, Gentili M, Callebaut I, Maschalidi S, Stolzenberg MC, Goudin N, Fremond ML, Nitschke P, Molina TJ, Blanche S, Picard C, Rice GI, Crow YJ, Manel N, Fischer A, Bader-Meunier B and Rieux-Laucat F. Inherited STING-activating mutation underlies a familial inflammatory syndrome with lupus-like manifestations. *J Clin Invest*. 2014;124:5516-20.

2. Konno H, Chinn IK, Hong D, Orange JS, Lupski JR, Mendoza A, Pedroza LA and Barber GN. Pro-inflammation Associated with a Gain-of-Function Mutation (R284S) in the Innate Immune Sensor STING. *Cell Rep*. 2018;23:1112-1123.

3. Liu Y, Jesus AA, Marrero B, Yang D, Ramsey SE, Sanchez GAM, Tenbrock K, Wittkowski H, Jones OY, Kuehn HS, Lee CR, DiMattia MA, Cowen EW, Gonzalez B, Palmer I, DiGiovanna JJ, Biancotto A, Kim H, Tsai WL, Trier AM, Huang Y, Stone DL, Hill S, Kim HJ, Hilaire CS, Gurprasad S, Plass N, Chapelle D, Horkayne-Szakaly I, Foell D, Barysenka A, Candotti F, Holland SM, Hughes JD, Mehmet H, Issekutz AC, Raffeld M, McElwee J, Fontana JR, Minniti CP, Moir S, Kastner DL, Gadina M, Steven AC, Wingfield PT, Brooks SR, Rosenzweig SD, Fleisher TA, Deng Z, Boehm M, Paller AS and Goldbach-Mansky R. Activated STING in a vascular and pulmonary syndrome. *N Engl J Med*. 2014;371:507-518.

4. Melki I, Rose Y, Uggenti C, Van Eyck L, Fremond ML, Kitabayashi N, Rice GI, Jenkinson EM, Boulai A, Jeremiah N, Gattorno M, Volpi S, Sacco O, Terheggen-Lagro SWJ, Tiddens H, Meyts I, Morren MA, De Haes P, Wouters C, Legius E, Corveleyn A, Rieux-Laucat F, Bodemer C, Callebaut I, Rodero MP and Crow YJ. Disease-associated mutations identify a novel region in human STING necessary for the control of type I interferon signaling. *J Allergy Clin Immunol*. 2017;140:543-552 e5.

5. Munoz J, Rodiere M, Jeremiah N, Rieux-Laucat F, Oojageer A, Rice GI, Rozenberg F, Crow YJ and Bessis D. Stimulator of Interferon Genes-Associated Vasculopathy With Onset in Infancy: A Mimic of Childhood Granulomatosis With Polyangiitis. *JAMA Dermatol*. 2015;151:872-7.

6. Omoyinmi E, Melo Gomes S, Nanthapisal S, Woo P, Standing A, Eleftheriou D, Klein N and Brogan PA. Stimulator of interferon genes-associated vasculitis of infancy. *Arthritis Rheumatol*. 2015;67:808.

7. Chia J, Eroglu FK, Ozen S, Orhan D, Montealegre-Sanchez G, de Jesus AA, Goldbach-Mansky R and Cowen EW. Failure to thrive, interstitial lung disease, and progressive digital necrosis with onset in infancy. *J Am Acad Dermatol*. 2016;74:186-9.

8. Picard C, Thouvenin G, Kannengiesser C, Dubus JC, Jeremiah N, Rieux-Laucat F, Crestani B, Belot A, Thivolet-Bejui F, Secq V, Menard C, Reynaud-Gaubert M and Reix P. Severe Pulmonary Fibrosis as the First Manifestation of Interferonopathy (TMEM173 Mutation). *Chest*. 2016;150:e65-71.

9. Saldanha RG, Balka KR, Davidson S, Wainstein BK, Wong M, Macintosh R, Loo CKC, Weber MA, Kamath V, Circa, Aadry, Moghaddas F, De Nardo D, Gray PE and Masters SL. A Mutation Outside the Dimerization Domain Causing Atypical STING-Associated Vasculopathy With Onset in Infancy. *Front Immunol*. 2018;9:1535.

10. Konig N, Fiehn C, Wolf C, Schuster M, Cura Costa E, Tungler V, Alvarez HA, Chara O, Engel K, Goldbach-Mansky R, Gunther C and Lee-Kirsch MA. Familial chilblain lupus due to a gain-of-function mutation in STING. *Ann Rheum Dis*. 2017;76:468-472.
