# Supplementary figures and images for: Novel TMEM173 Mutation and the Role of Disease Modifying Alleles
Source: Front Immunol. 2019 Dec 5;10:2770. doi: 10.3389/fimmu.2019.02770 (PMC6907089; doi:10.3389/fimmu.2019.02770)

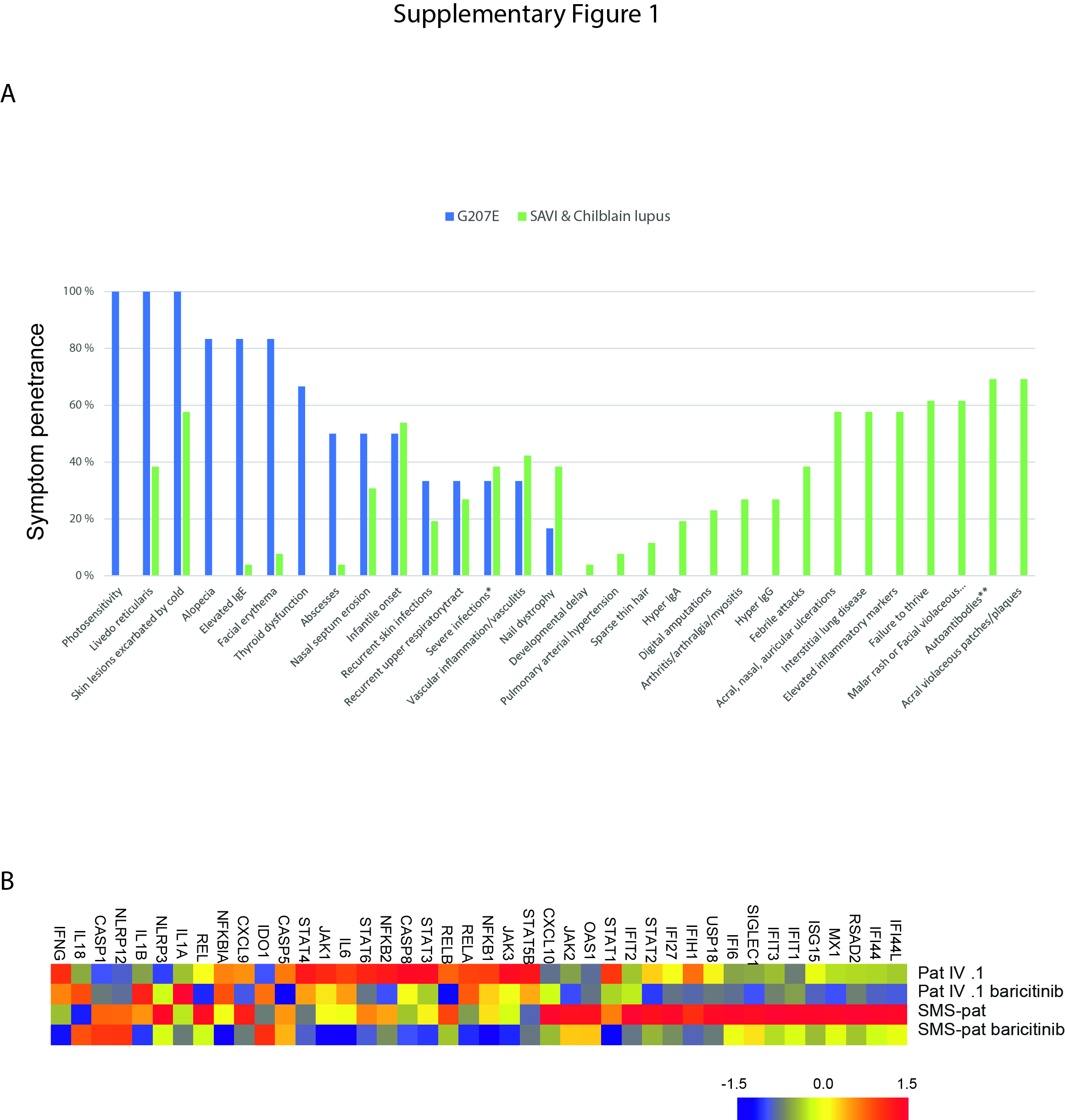

Supplement: Supplementary file 4 [file Image_1.JPEG]

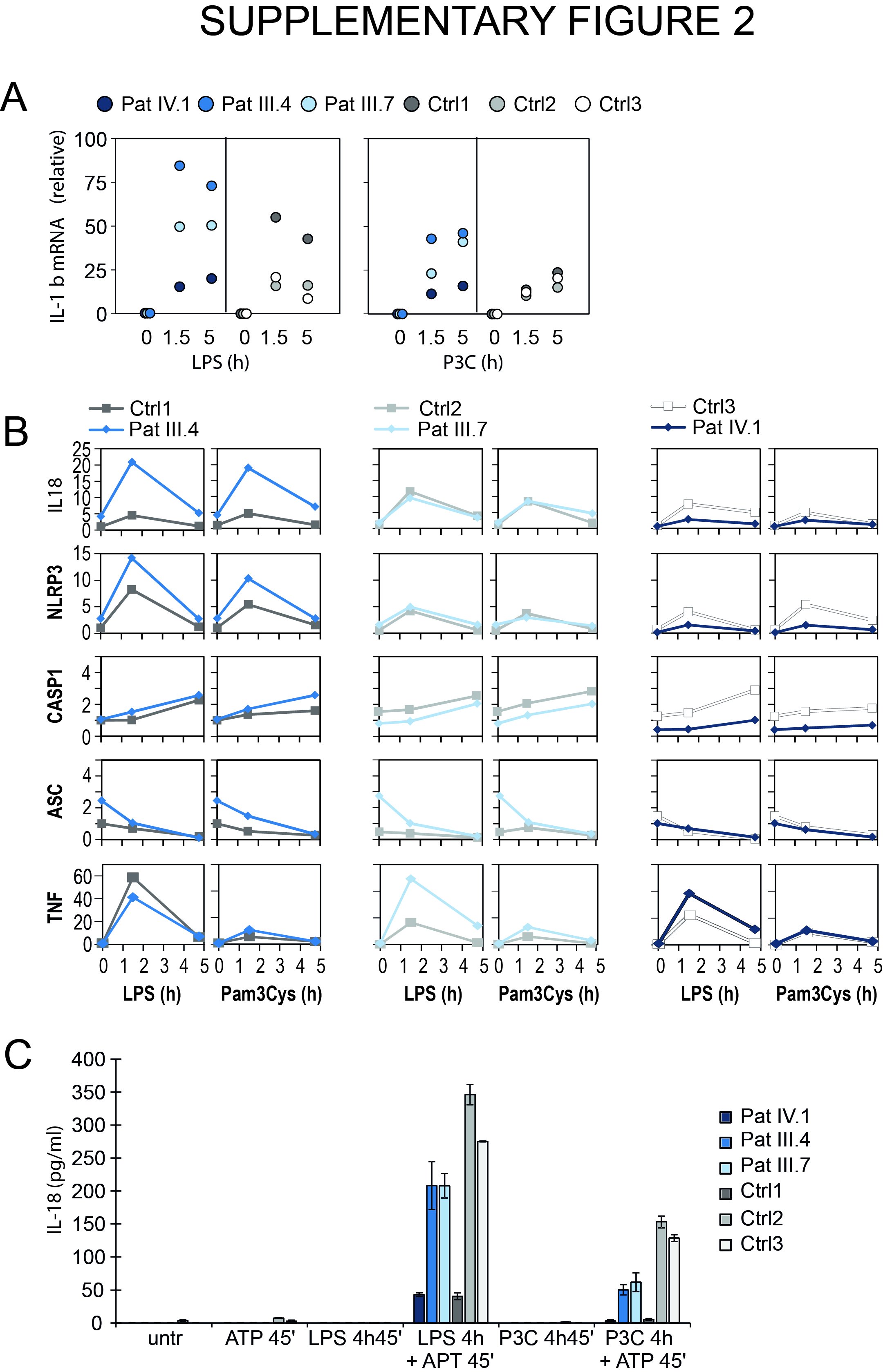

Supplement: Supplementary file 5 [file Image_2.JPEG]

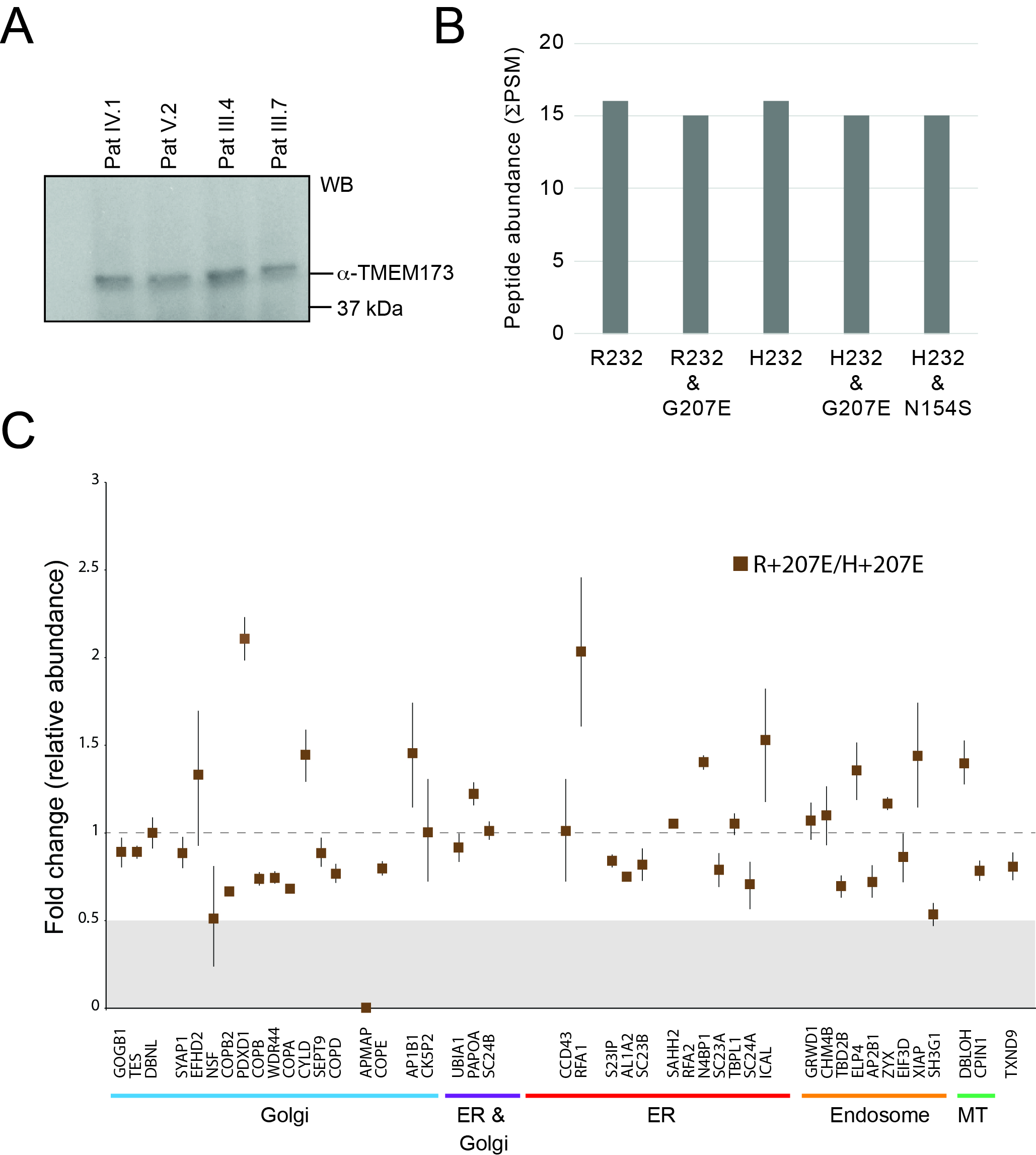

Supplement: Supplementary file 6 [file Image_3.JPEG]
